# The effect of hormone replacement therapy on cognitive function in postmenopausal women: An RCT

**DOI:** 10.18502/ijrm.v16i12.3682

**Published:** 2019-01-28

**Authors:** Fereshteh Moradi, Shahideh Jahanian Sadatmahalleh, Saeideh Ziaei

**Affiliations:** Department of Midwifery and Reproductive Health, Faculty of Medical Sciences, Tarbiat Modares University, Tehran, Iran.

**Keywords:** *Cognition*, * Hormone replacement therapy*, * Postmenopausal women.*

## Abstract

**Background:**

During the reproductive age, the human brain becomes a target for gonadal steroid hormones. Estrogens influence neural function through effects on neurons and affects indirectly the oxidative stress, inflammation, the cerebral vascular and the immune system.

**Objective:**

To evaluate the effect of the traditional hormone replacement therapy (HRT) on the cognitive function in postmenopausal women.

**Materials and Methods:**

In this randomized clinical trial, 140 postmenopausal women, from November 2014 to February 2015, were included. Women were randomly divided into two groups. Each woman in the case group took traditional HRT (0.625mg conjugated equine estrogens+2.5mg medroxyprogesterone acetate daily) plus one Cal+D tablet (500 mg calcium+200 IU vitamin D) daily for four months. Women in the control group received only one Cal+D tablet (500 mg calcium+200 IU vitamin D) daily for four months period. The Montreal Cognitive Assessment (MoCA) and Green Climacteric Scale (GCS) questionnaires filled out after the intervention and compared between the two groups.

**Results:**

The mean points of the MoCA after the intervention indicate that all MoCA domains except for the orientation improved in the case group. There was a significant difference in the memory domain after the treatment between the two groups. MoCA domains and GCS were negatively correlated after the intervention (r=-0.235,p=0.006).

**Conclusion:**

The HRT has affected some of the MoCA factors. The effects of HRT on cognitive function should be studied in a large prospective study in a group of women in their early and late menopausal ages with periodic assessment of their cognitive function during these follow-up years.

## 1. Introduction

During the reproductive age, the human brain becomes a target for gonadal steroid hormones (1). Estrogens influence neural function through effects on neurons and effects indirectly on oxidative stress, inflammation, the cerebral vascular and the immune system (2-5). Forgetfulness is common during postmenopausal age, and many women complain of cognitive and emotional problems at times that are associated with decreasing in circulating levels of estrogens. Some researchers have suggested that declines in estrogen level may lead to deficits in the cognitive ability, including memory, attention, language and visuospatial ability, of post-menopausal women (6–8). Mild cognitive impairment (MCI) is episodic memory loss without dementia. Approximately 15 to 20 percent of people age 65 or older have MCI. However, MCI does not always lead to dementia, the rate of transition from MCI to dementia is 10–20% per year (4, 9, 10).

Some evidence from clinical trials in postmenopausal women especially in older ones suggested that hormone replacement therapy (HRT) has no substantial cognitive effect (11–13). However, for surgically menopausal women, Ratka and co-workers found that estrogen therapy could be of cognitive benefit (14). In women with menopausal symptoms during the early postmenopause, HRT may have specific cognitive effects, and future research should target these effects. Postmenopausal women who experience more hot flashes, particularly while sleeping, have a better cognitive function than postmenopausal women who did not experience hot flashes, according to a pilot study (15).

The objective of the present study was to evaluate the effect of the traditional HRT on the cognition of postmenopausal women through a randomized controlled study. Also, the authors evaluate the relationship between cognitive function and climacteric symptoms in postmenopausal women after the traditional HRT.

## 2. Materials and Methods

**Figure 1:**
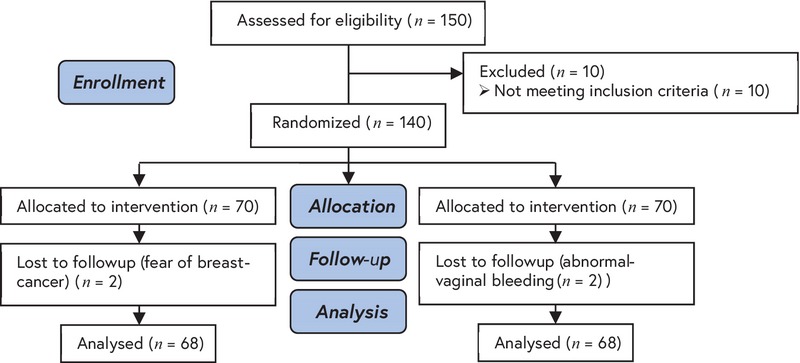
The consort flowchart.

This randomized clinical trial was conducted on 140 postmenopausal women (Figure 1), who attended Gynecology Clinic in Arash Hospital in Tehran, Iran from November 2014 to February 2015.

The inclusion criteria were women whose menstruation had ceased at least 1 yr before and not more than 10 yr, age between 50 and 60 yr, and with serum Follicle-stimulating hormone concentrations over 40 Iu/ml. Volunteers had not undergone hormone therapy during the six months prior to the trial. All subjects were urban. Women suffering from chronic diseases such as diabetes, hypertension, cardiovascular diseases, psychiatric diseases, cancer and those who smoke were deemed ineligible. At the time of enrollment, the interviewer administered a questionnaire to collect baseline information on the sociodemographic status and medical history. Then, participants filled out Montreal Cognitive Assessment (MOCA) and Green Climacteric Scale (GCS) questionnaire for evaluation of cognitive function status and climacteric symptoms, respectively.

Women were randomly divided into two groups. Randomization was performed through computer-generated list of random number groups. Each woman in the case group took traditional HRT (0.625 mg conjugated equine estrogens plus 2.5 mg medroxyprogesterone acetate daily) plus one Cal+D tablet (500 mg calcium+200IU vitamin D) daily for four months. Women in the control group received only one Cal+D tablet (500 mg calcium+200IU vitamin D) daily for four-months period. The MoCA and GCS were assessed after the intervention and compared between the two groups. Also, the relationship between cognitive function and climacteric symptoms was evaluated after the interventions. The MoCA was created and validated by Nasreddine and colleagues (15).

The MoCA is a brief 30-question test that takes around 10–12 minutes to complete. Scores on the MoCA range 0–30, with a score of 26 and higher generally considered normal, while scores less than 26 are abnormal and suggestive of developing mild cognitive impairment (MCI) The MoCA assesses multiple cognitive domains including visuospatial and executive functioning (5 points), animal naming (3 points), attention (6 points), language (3 points), abstraction (2 points), delayed recall (short-term memory) (5 points), orientation (6 points), education level (1 point is added to the test-taker's score if he or she has 12 years or less of formal education) (15).

Psychometric properties of this scale have been studied in several studies and its validity and reliability have been confirmed (16). The GCS is a self-report measure for menopausal symptoms. The GCS contains 21 items divided into various clusters with individual values. The clusters are psychological (11 symptoms) subdivided into anxiety and depression, somatic (7 symptoms), vasomotor (2 symptoms) and sexual (1 symptom). Each symptom is rated according to its severity using a four-point Likert scale (0, not at all; 1, a little; 2, quite a bit; 3, extremely). The GCS is the sum of all 21 scores ranging from 0 to 63. A higher total score corresponds with more menopausal symptoms (17).

### Ethical consideration

This study was conducted with the approval of the Ethics Committee of Tarbiat Modares University of Medical Sciences (IRB#525000). All the women were informed about the project and had given a written consent before participating in the study.

### Statistical analysis

Based on the results of a pilot study, with α=0.05 and β=0.2, the sample size for two independent samples was calculated as 65 for each arm. Thus, to allow for loss to follow up, 70 consecutive postmenopausal women who had inclusion criteria were eligible in this trial. The data of normality in distribution was examined by K-S. As distribution was assumed to be normal in all variables, independent *T*-test, Paired *T*-test, Chi-square test and the Pearson Correlation test was applied; p<0.05 was considered statistically significant. All statistical tests were 2-tailed. All statistical analyses were performed using the SPSS software (Statistical Package for the Social Sciences, version 20.0, SPSS Inc, Chicago, IL, USA).

## 3. Results

In a randomized, controlled trial, 140 non-surgically postmenopausal women were allocated into three groups. Four women were excluded from the initial analyses, fear of breast cancer in two women and abnormal vaginal bleeding in two other women. Finally, 68 cases and 68 controls completed the study and analyzed. (Figure 1). There were no significant differences in demographic and clinical characteristics between the two groups after the randomization (Table I). At baseline, there were no significant differences in all domains of MoCA and Greene climacteric scale between the two groups (Table II). The mean points of the MoCA after the intervention indicate that all MoCA domains except for the orientation improved in the case group and not in the control group. There was a significant difference in the memory domain after the treatment between the two groups (Table III). Also, as it is shown in Table III, MoCA domains and Greene climacteric scales were negatively correlated after the intervention (r=-0.235p=0.006).

**Table 1:** Comparison of demographic characteristics between the two groups.


	**HRT group (n: 68)**	**Control group (n: 68)**	**p-value**
Women age (yr)*		55.97 ± 4.92	55.18 ± 4.72	0.339
BMI*		26.35 ± 4.33	26.88 ± 4.19	0.470
Duration from last menses (Yr)*		5.65 ± 2.61	5.17 ± 2.73	0.292
Parity*		2.24 ± 1.57	2.10 ± 1.49	0.610
Educational status**				
	Basic school	24 (35.3)	17 (25)	0.119
	High school and university	44 (64.7)	51 (75)	
Occupation **				
	Housewife	33 (48.5)	37 (54.4)	0.494
	Employer	35 (51.5)	31 (45.6)	

ndependent Student *t*-test (Mean ± SD).

** Chi square (n: %)

BMI: Body Mass Index

HRT: Hormone replacement therapy

**Table 2:** Comparison between the two groups in MoCA points and GCS scores before and after the treatment (Mean ± SD).


	**HRT group (n: 68)**		**Control group (n: 68)**		**p-value a**	**p-value b**	**p-value c**	**p-value d**
	**Before**	**After**	**Before**	**After**		
Visuospatial/Executive	5.91 ± 0.99	6.24 ± 1.01	5.96 ± 0.94	5.97 ± 1.02	0.795	0.820	< 0.001	0.131
Memory	3.06 ± 0.75	3.65 ± 0.95	3.25 ± 0.79	3.24 ± 0.83	0.153	0.568	< 0.001	0.008
Attention	4.72 ± 0.89	5.03 ± 0.86	4.92 ± 0.96	4.88 ± 0.92	0.200	0.495	< 0.001	0.519
Language	2.24 ± 0.75	2.43 ± 0.74	2.25 ± 0.76	2.28 ± 0.73	0.910	0.159	< 0.001	0.245
Abstraction	1.47 ± 0.72	1.66 ± 0.56	1.59 ± 0.67	1.57 ± 0.72	0.328	0.568	< 0.001	0.427
Orientation	5.26 ± 1.02	5.31 ± 0.96	5.37 ± 0.99	5.35 ± 1.03	0.551	0.658	0.536	0.797
Total GCS score	22.82 ± 7.48	18.76 ± 5.61	21.75 ± 8.38	21.37 ± 8.21	0.431	0.135	< 0.001	0.033
Total MoCA score	22.75 ± 2.69	24.26 ± 2.98	23.35 ± 2.95	23.38 ± 2.98	0.373	0.805	< 0.001	0.483

*Independent student *t*-test paired *T*-test

a: Between the two groups before the peatment b: Before and after the intervention in conpol group

c: Before and after the peatment in HRT group d: Between the two groups after the peatment

GCS: Green Climacteric Scale MoCa: Monpeal Cognitive Assessment HRT: Hormone Replacement Therapy

**Table 3:** Correlation of MoCA points and GCS scores after the intervention (*n*: 138).*


	**Total GCS scores**	**Psychological scores**	**Somatic scores**	**Vasomotor scores**	**Sexual scores**
Total MoCA	r:–0.235	r:–0.127	r:–0.182	r:–0.176	r:–0.176
Points	P:0.006	P:0.139	P:0.034	P:0.041	P:0.042

*: Pearson correlation

MoCA: Montreal cognitive assessment GCS: Green climacteric scale

## 4. Discussion

The mean points of the MoCA after the intervention indicate that all MoCA domains, except for the orientation improved in the case group. There was a significant difference in the memory domain after the treatment between the two groups. MoCA domains and Greene climacteric scales were negatively correlated after the intervention.

A majority of menopausal women report cognitive dysfunction. Two pathways may underline menopause-associated alternation in cognitive function. 1) A symptom associated with the menopause may cause poorer cognitive performance 2) estrogen may directly benefit neural tissue; estrogen augments hippocampal and prefrontal cortical function, potentially enhancing verbal memory and executive function. Reduction in estrogen levels during menopause could therefore negatively affect cognitive function. Then, hormone therapy with amelioration of vasomotor symptoms may improve cognitive impairment (3).

This study had two aims. The first was to investigate whether HRT improves cognitive performance in postmenopausal women. The second was to consider the relationship between menopause-associated symptoms with cognitive function. The results of this study show improvement in cognitive function, memory, visuospatial/executive, attention, language and abstraction domains after the HRT treatment. However, in comparison with the control group, only memory domain improves after HRT. These findings have been replicated in Ratca and colleagues' study (14). Although the current finding is not consistent with other reports (12, 13, 18, 19), Fischer *et al*. showed an equivocal regarding the benefits of HRT to cognition and affect (20). The other results suggest that HRT when taken early postmenopause may have a beneficial effect on cognitive control prefrontal mechanisms (21).

The WHI study showed that HRT (CEE/MPA) worsened verbal memory and dementia risk in women aged 65 yr and above, but not the risk of mild cognitive impairment (12). Differences in the test used to assess cognitive function, assessing dementia and/or mild cognitive impairment, as well as the difference in mean age might explain these discrepancies. Considering these conflicting results in the literature, further research based on well-controlled clinical trials with large sample is necessary to yield a consistent conclusion. The authors used MoCA for evaluation of cognitive function. The MoCA has been tested and validated in the setting of MCI and has subsequently been adopted in numerous other settings clinically. It was completed in 10 min. This test was recently proposed as a cognitive screening test for MCI, having surpassed the well-known limitations of the Mini-Mental State Examination (MMSE). The MMSE had a sensitivity of 18% to detect MCI, whereas the MoCA detected 90% of MCI subjects. Specificity was excellent for both MMSE and MoCA (100% and 87%, respectively).The authors found that MOCA domains negatively correlated with Greene climacteric scales.

Lower perfusion in specific areas of the cerebral cortex correlated significantly with rapid deterioration in the MMSE. The decline in gonadal steroids hormones during menopause gives rise to a wide range of physiological and psychological As a result of treatment with estrogens, there was an increased global blood flow (2–4). One pilot study reported that the cognitive function of women who had hot flash was better than in women who did not (15). It is hypothesized that hot flashes in postmenopausal women a counter-regulatory response to the impaired of glucose delivery to the brain and may have a beneficial effect on general cognitive function. This hypothesis was discussed by Ratka (14) who proposed two possible pathways of immediate and delayed responses. "In the first pathway, in the absence of a counter-regulatory mechanism and lack of hot flashes, a cascade of neuropathological reactions evoked by glucose deprivation in the brain is initiated. It is likely that a reduced carbohydrate metabolism in the brain forms a basis for other processes known to cause neuronal injury and possible cognition impairment. In the second possible pathway, in response to glucose deprivation in the brain, a hot flash occurs that triggers a counter-regulatory mechanism aimed at the delivery of glucose to the brain and also a critical signal initiating a brain-defense mechanism to counter-regulate the cascade of detrimental neurological processes."

## 5. Conclusion

The HRT has affected some of the MoCA factors. The relationship between climacteric symptoms and cognitive function should be studied in a large prospective study in a group of women in their early and late menopausal ages with periodic assessment of their cognitive function during these follow-up years.

##  Conflict of Interest

The authors have no conflicts of interest.

## References

[1] Nguyen T.-V., Ducharme S., Karama S. (2017). Effects of Sex Steroids in the Human Brain. *Molecular Neurobiology*.

[2] Sturdee D. W., Pines A. (2011). Updated IMS recommendations on postmenopausal hormone therapy and preventive strategies for midlife health. *Climacteric*.

[3] Greendale G. A., Wight R. G., Huang M.-H., Avis N., Gold E. B., Joffe H., Seeman T., Vuge M., Karlamangla A. S. (2010). Menopause-associated symptoms and cognitive performance: Results from the study of women's health across the nation. *American Journal of Epidemiology*.

[4] Etgen T., Sander D., Bickel H., Förstl H. (2011). Mild Cognitive Impairment and Dementia. *Deutsches Aerzteblatt Online*.

[5] Karlamangla A. S., Lachman M. E., Han W., Huang M., Greendale G. A. (2017). Evidence for cognitive aging in midlife women: Study of Women's Health Across the Nation. *PLoS ONE*.

[6] Pimenta F., Leal I., Maroco J., Ramos C. (2012). Menopausal symptoms: Do life events predict severity of symptoms in peri- and post-menopause?. *Maturitas*.

[7] Goveas J. S., Espeland M. A., Hogan P., Dotson V., Tarima S., Coker L. H., Ockene J., Brunner R., Woods N. F., Wassertheil-Smoller S., Kotchen J. M., Resnick S. (2011). Depressive symptoms, brain volumes and subclinical cerebrovascular disease in postmenopausal women: The Women's Health Initiative MRI Study. *Journal of Affective Disorders*.

[8] Goveas J. S., Espeland M. A., Woods N. F., Wassertheil-Smoller S., Kotchen J. M. (2011). Depressive symptoms and incidence of mild cognitive impairment and probable dementia in elderly women: The women's health initiative memory study. *Journal of the American Geriatrics Society*.

[9] Rapp S. R., Legault C., Henderson V. W., Brunner R. L., Masaki K., Jones B., Absher J., Thal L. (2010). Subtypes of mild cognitive impairment in older postmenopausal women: The women s health initiative memory study. *Alzheimer Disease & Associated Disorders*.

[10] Mitchell E. S., Woods N. F. (2011). Cognitive symptoms during the menopausal transition and early postmenopause. *Climacteric*.

[11] Etgen T., Bickel H., Förstl H. (2010). Metabolic and endocrine factors in mild cognitive impairment. *Ageing Research Reviews*.

[12] Maki P. M., Henderson V. W. (2012). Hormone therapy, dementia, and cognition: The Women's Health Initiative 10 years on. *Climacteric*.

[13] Scali J., Ryan J., Carrière I., Dartigues J.-F., Tavernier B., Ritchie K., Ancelin M.-L. (2010). A prospective study of hormone therapy and depression in community-dwelling elderly women: The three city study. *Journal of Clinical Psychiatry*.

[14] Ratka A. (2005). Menopausal hot flashes and development of cognitive impairment. *Annals of the New York Academy of Sciences*.

[15] Nasreddine Z. S., Phillips N. A., Bédirian V., Charbonneau S., Whitehead V., Collin I., Cummings J. L., Chertkow H. (2005). The Montreal Cognitive Assessment, MoCA: a brief screening tool for mild cognitive impairment. *Journal of the American Geriatrics Society*.

[16] Emsaki G., Molavi H., Chitsaz A., Abtahi M. M., Asgari K. (2011). Psychometric properties of the montreal cognitive assessment (MoCA) in Parkinson’s disease patients in Isfahan. *Journal of Isfahan Medical School*.

[17] Greene J. G. (1976). A factor analytic study of climacteric symptoms. *Journal of Psychosomatic Research*.

[18] Henderson V. W., St John J. A., Hodis H. N., McCleary C. A., Stanczyk F. Z., Shoupe D., Kono N., Dustin L., Allayee H., Mack W. J. (2016). Cognitive effects of estradiol after menopause. *Neurology*.

[19] Gleason C. E., Dowling N. M., Wharton W., Manson J. E., Miller V. M., Atwood C. S., Brinton E. A., Cedars M. I., Lobo R. A., Merriam G. R., Neal-Perry G., Santoro N. F., Taylor H. S., Black D. M., Budoff M. J., Hodis H. N., Naftolin F., Harman S. M., Asthana S., Brayne C. (2015). Effects of Hormone Therapy on Cognition and Mood in Recently Postmenopausal Women: Findings from the Randomized, Controlled KEEPS–Cognitive and Affective Study. *PLoS Medicine*.

[20] Fischer B., Gleason C., Asthana S. (2014). Effects of hormone therapy on cognition and mood. *Fertility and Sterility*.

[21] Girard R., Météreau E., Thomas J., Pugeat M., Qu C., Dreher J. (2017). Hormone therapy at early post-menopause increases cognitive control-related prefrontal activity. *Scientific Reports*.

